# Newborn Pulse Oximetry Screening for Detecting Congenital Heart Disease: Experience at a Tertiary Care Center

**DOI:** 10.1155/2024/3279878

**Published:** 2024-01-11

**Authors:** Ziad R. Bulbul, Nour K. Younis, Farah Malaeb, Haytham Bou Hussein, Mariam Arabi, Fadi Bitar

**Affiliations:** ^1^Department of Pediatrics, American University of Beirut Medical Center, Beirut, Lebanon; ^2^Faculty of Medicine, American University of Beirut Medical Center, Beirut, Lebanon

## Abstract

**Background:**

Congenital heart disease (CHD) remains the number one birth defect worldwide. Pulse oximetry screening (POS) is a widely used CHD screening modality effective in detecting critical lesions. This study is aimed at assessing the accuracy and cost-effectiveness of POS in a cohort of term well-babies admitted to a regular nursery in a tertiary care center.

**Methods:**

We reviewed the charts of term babies admitted to our regular nursery over a period of one year. The results of POS and the findings of echocardiography were collected. Similarly, we explored the records of our fetal echocardiography program to identify the fetuses screened for CHD during the same period.

**Results:**

900 term babies were born and admitted to newborn nursery at our center, and 69 fetuses were evaluated by our fetal cardiology team during the study period. None of our term babies had a positive POS at birth or 24 hours of age. However, 56 babies had a cardiac echo before hospital discharge due to suspicious findings on physical examination or a family history of CHD. A simple noncritical CHD was noted in 10 of them. Additionally, 53 babies underwent echocardiography within the first five years of life; a simple CHD was noted in 6 of them. In parallel, 21 of our fetuses were found to have CHD: 16 simple CHD and 5 critical CHD (CCHD).

**Conclusion:**

Despite its cost-effectiveness and efficacy in screening for CCHD, POS is suboptimal for detecting simple CHD. In the absence of a proper prenatal screening and fetal echocardiography program, POS remains a cost-effective modality for detecting CCHD.

## 1. Introduction

CHD is the most encountered congenital defect worldwide [1]. It afflicts millions of living individuals and has an estimated annual incidence of around 1%. CHD prevalence may vary from one country to the other depending on the availability and efficacy of pre- and post-natal screening [2–4]. In the absence of the appropriate screening tools, many asymptomatic mild types of CHD remain undetected and may be, thus, underreported. Moreover, even when adequate prenatal sonography and clinical assessment are performed, up to 25% of CHD cases are missed [5]. This suggests that additional more sensitive and specific tools are needed to ensure proper and early detection of CHD.

Furthermore, around 50% of all CHD patients are asymptomatic early in life [6]. However, approximately half of the remaining patients will require correction or palliation using catheter or surgical interventions during the first year of life [7]. In patients with a ductal-dependent defect (complex lesions in whom survival depends on the patency of the arterial duct, like d-transposition of the great arteries, and hypoplastic left heart syndrome), an early and timely diagnosis is of paramount importance in order to initiate measures to maintain the ductal patency and prepare for intervention [6]. Consequently, several tools have been employed to detect critical CHD (CCHD), particularly ductal-dependent lesions, requiring immediate intervention. Among these tools, pulse oximetry is deemed an accessible, noninvasive, rapid, and reliable tool that is widely applied in screening for CCHD at birth [8]. Pulse oximetry is also a cost- and time-effective screening method that can be easily acquired and applied in most countries.

The sensitivity and specificity of pulse oximetry were estimated at 100% and 99% by a couple of studies [6, 9]. However, in a more recent meta-analysis, its approximated sensitivity and specificity were 69% and 99%, respectively [10]. Similarly, in a recent Cochrane systematic review published in 2018 and involving 21 cross-sectional and cohort clinical studies, the sensitivity and specificity of this test were computed at 76.3% and 99.9%, respectively [11]. Hence, it is suggested that pulse oximetry is a moderately sensitive and a highly specific screening test for CCHD. Here, in this study, we aim to assess the effectiveness of this tool in screening for CHD, including both simple and critical CHD, at a tertiary care center in a developing country. We aspire as well to evaluate the prevalence of later-detected CHD among the studied cohort.

## 2. Study Aim

In this study, we aim to examine the effectiveness of pulse oximetry in screening for CHD in a tertiary care center with an active prenatal screening program. We aspire also to assess the benefit of performing routine pulse oximetry to all term babies admitted to nursery units and to evaluate the prevalence of a future diagnosis of CHD among a cohort of neonates previously screened for CHD using pulse oximetry.

## 3. Materials and Method

### 3.1. Study Design and Population

In our descriptive retrospective observational study, we included newborns admitted to the nursery unit at our tertiary care center, the American University of Beirut Medical Center (AUBMC), over a one-year period (Jan. 1, 2015-Dec. 31, 2015). We excluded (1) neonates who were born during this period but transferred directly to the neonatal intensive care unit for noncardiac causes and (2) neonates who had no documented oxygen saturation values either at birth or at 24 hours of age.

### 3.2. Data Collection and Analysis

After we received approval from the AUBMC Institutional Review Board (IRB), we reviewed charts of the eligible subjects and retrieved the following parameters: newborn gestational age and sex, family history of CHD, oxygen saturation at birth and at least 24 hours of age taken from the right upper extremity and either the right or left lower extremity, physical (particularly cardiac) exam findings, and echocardiography results (whenever performed).

We identified the total number of studied subjects and computed the difference between their pre- and post-ductal oxygen saturations. We assessed the prevalence of diagnosed CHD as well among these patients at birth and later during the first years of life. We classified CHD as per the tool of diagnosis into (1) CHD diagnosed using pulse oximetry at birth and (2) CHD suspected by physical exam and diagnosed using echocardiography.

During the same period, we examined the records of our fetal echocardiography program to estimate the incidence of prenatally detected CHD. We reviewed the charts of newborns who underwent fetal echocardiography during this year. The incidence of prenatally detected CHD was computed and compared to the postnatal incidence of CHD diagnosed using POS and postnatal echocardiography.

Over a period of one year, 900 term babies were born at our center and transferred to the regular nursery. Both pregnancy and delivery were smooth and uncomplicated. Hence, none of these babies had required a planned admission to the neonatal intensive care unit. Similarly, all had a prenatal ultrasound by obstetrician, and none was referred to our fetal cardiac center. All subjects were screened for CCHD using the pulse oximetry screening protocol recommended by the American Academy of Pediatrics (AAP). Pre- and post-ductal oxygen saturations were measured at birth and 24 hours of age using a pulse oximeter. The preductal oxygen saturation was taken from the right upper extremity, and the postductal saturation was measured either from the right or left lower extremities.

## 4. Results

**Figure 1:**
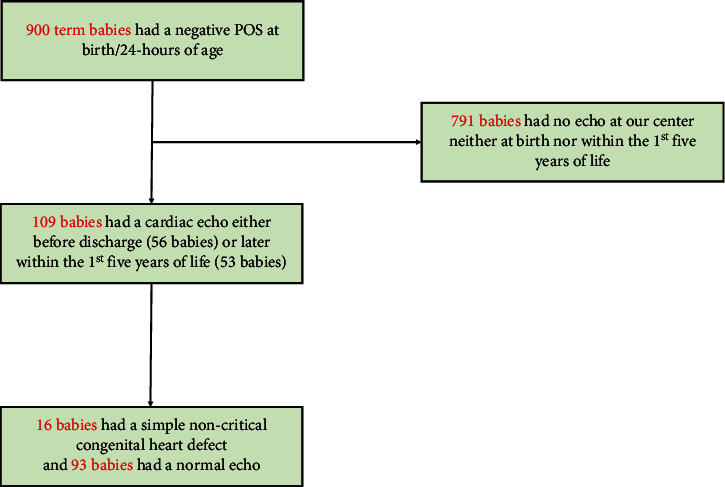
Schematic diagram of the cohort of term babies. 900 term babies were born at our center over a one-year period. A cardiac echo was offered to 109 babies either before discharge from nursery or within the first five years of life. A simple noncritical CHD was noted in 16 babies.

At birth, none of our newborns had a pre- or post-ductal oxygen saturation of less than 95% (Figure 1). 662 newborns (73.56%) had an SpO_2_ of 100%, 157 (17.44%) had an SpO_2_ of 99%, and 65 (7.22%) newborns had an SpO_2_ of 98%. An SpO_2_ of 95% to 97% was noted in the remaining sixteen babies (less than 2%) (Table 1). There was no difference between the pre- and post-ductal SpO_2_ in any of our babies. Similarly, at 24 hours of age, an SpO_2_ of 100% was noted in most of the babies (76%). SpO_2_ was 99% and 98% in 146 (16.22%) and 56 (6.22%) babies, respectively. The rest, 14 babies (<2%), had an SpO_2_ of 95% to 97%. No difference was detected between the pre- and post-ductal SpO_2_ in any of these babies at 24 hours of age as well.

Within the first days of life, 56 babies had undergone a cardiac echo before discharge due to an abnormal finding on physical examination (i.e., murmur, single umbilical artery, and single palmar crease), a family history of CHD, an echogenic focus on fetal ultrasound, an abnormal fetal electrocardiogram, or an abnormal laboratory finding like direct hyperbilirubinemia (see Table 2). Out of these babies, 41 (73.2%) were found to have a murmur on cardiac examination and had then an echocardiography to rule out CHD. Three (5.4%) had a family history of CHD, and three (5.4%) had an echogenic focus noted on fetal ultrasound.

As for the echo findings, a ventricular septal defect (VSD) was observed in seven babies, an atrial septal defect (ASD) in two, and a bicuspid aortic valve (BAV) in one. Age-expected findings like patent ductus arteriosus (PDA), patent foramen ovale (PFO), and peripheral pulmonary stenosis (PPS) were noted in the majority of these 56 babies. PDA, PFO, and PPS were observed in 43, 20, and 16 babies, respectively. Twenty-three babies had at least one visit at our cardiac center between 2015 and 2020, around five years after birth. At that time, six patients had spontaneous closure of VSD, and one was left with a tiny residual VSD.

An additional 53 subjects had an echocardiography within the first five years of life. They were referred to our cardiac center for the following reasons: fatigue, syncope, cyanosis, a murmur noted on exam, a suspected Kawasaki syndrome, or a suspected perimyocarditis. Forty-seven patients (89%) had a normal cardiac echo, and six (11%) were found to have an ASD requiring intervention, including one of the sinus venosus type. In summary, no critical congenital cardiac defects were diagnosed in the 109 babies (12% of our cohort) who underwent echocardiography either at birth or within the first five years of life. Sixteen patients were found to have a noncritical CHD like ASD, VSD, or BAV.

**Figure 2:**
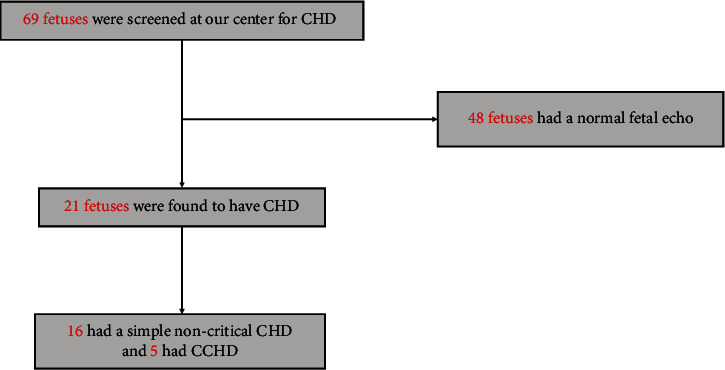
Schematic diagram of the cohort of fetuses referred for fetal echocardiography. 69 fetuses were offered a fetal echo at our center. 21 were diagnosed with a CHD; 16 had a simple CHD, and 5 had a critical CHD.

Over the same period, 69 pregnant women were referred to our cardiac center for fetal echocardiography (Figure 2). Most of these patients (48/69) were followed up by obstetricians at our tertiary care center. The rest (21/69) were referred from different national and regional primary or secondary medical centers. 21 fetuses were diagnosed with CHD, of whom five were diagnosed to have a CCHD (one with tricuspid atresia, one with single ventricle and malposed great arteries, one with TGA, one with hypoplastic left heart syndrome, and one with mitral atresia, single ventricle, and hypoplastic aorta), see Table 3. Ten of these fetuses (48%) were delivered at our specialized tertiary care center and transferred immediately to the neonatal intensive care unit (NICU). Of these 69 fetuses, 38 had a negative fetal echo and were delivered at our center and transferred to regular nursery. Given this, our cohort was composed of 900 babies and 31 fetuses.

## 5. Discussion

CHD is the most frequently diagnosed congenital defect. It affects an estimate of around 1 per 100 live births. The prevalence of CHD is impacted by multiple socioeconomic and medical factors. These factors include and are not limited to the presence of efficacious screening techniques, the availability of skilled obstetricians and pediatric cardiologists, the level of parental education and socioeconomic status, and the existence of parental consanguinity. For instance, in a previous study performed at our center, the incidence of CHD was estimated at 1.15% [4]. This incidence was higher than the global incidence of CHD estimated at 0.8 to 1%. This difference was attributed partly to parental consanguinity that is particularly prevalent in our society. Additionally, many of the encountered lesions were simple CHD-like VSD and pulmonary stenosis. The incidence of complex CHD was comparatively minimal [4].

CHD screening is not limited to postnatal modalities like pulse oximetry, neonatal physical examination, and echocardiography. It begins prenatally and can be achieved using a fetal ultrasonography performed by a skilled obstetrician. A detailed fetal ultrasound portraying the four cardiac cavities and the outflow tracts is required to assess the heart's anatomy and physiology and, thus, to identify underlying CHD. Questionable or positive screenings are then referred to a fetal echocardiographer for confirmation and, whenever necessary, planning of delivery. This regularly applied modality is not properly performed in all countries. Congruently, the sensitivity and specificity of this modality in detecting simple and critical CHD vary markedly from one country to another and even from one city/state to another [7, 12–14]. Moreover, most data are extrapolated from clinical studies conducted in developed countries. Perhaps, in the United States of America, the overall annual rate of prenatal CHD screening was estimated at 34% as per a large cross-sectional study published in 2015 [13]. More recently, as per an international cohort study published in 2019, the overall rate of critical CHD (CCHD) prenatal detection was estimated at 50% [7]. In this study, more than 8 million neonates were included, and CCHD was observed only in 18,243 babies. The rates of prenatal detection of these defects were between 13% and 87%, with the highest being in France and the lowest in the Slovak Republic [7]. This indicates that prenatal CHD screening is not equally effective in all nations and suggests that other simpler modalities, such as POS, are needed to screen for CHD, particularly CCHD.

**Figure 3:**
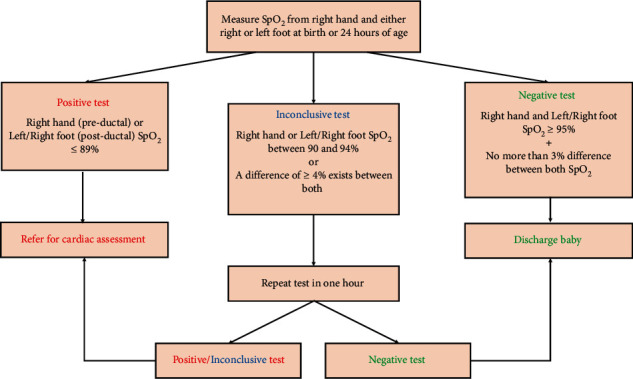
Pulse oximetry screening algorithm.

Pulse oximetry is a widely available inexpensive tool used in screening for ductal-dependent CHD in newborns. This tool is often the sole screening modality for CHD offered to term well-appearing babies admitted to regular nurseries. As mentioned previously, POS is proven to be a moderately sensitive and highly specific CCHD screening modality. However, POS is not optimal for detecting noncritical CHD as they display limited to no hypoxemia. POS involves measuring pre- and post-ductal SpO_2_ from the right hand and a lower extremity, respectively [15]. A positive screening test, represented by a pre- or post-ductal SpO_2_ of 89% or less, warrants prompt assessment using echocardiography. POS is considered negative if pre- and post-ductal SpO_2_ are at least 95% and are not different by more than 3% [15]. POS is inconclusive if (1) pre- or post-ductal SpO_2_ is between 90 and 94% or (2) a difference of at least 4% exists between pre- and post-ductal SpO_2_. An inconclusive test should be repeated after one hour; a repeat negative POS is satisfactory. A repeat inconclusive or positive POS necessitates further cardiac assessment to rule out CCHD (see Figure 3).

Here, in this retrospective observational study, we examined the accuracy of POS in detecting CCHD. A total of 900 term babies and 31 fetuses were included. None of our term babies had a positive POS. However, 109 babies were evaluated by our pediatric cardiologist either at birth or within the 1^st^ five years of life (Figure 1). They underwent a cardiac echo due to various reasons, as mentioned in Table 2. The presence of a murmur on physical examination was the leading cause of CHD screening in these babies. Similarly, a family history of CHD, peripheral cyanosis, and an abnormal fetal morphology scan were among the most important reasons for CHD screening. Of these 109 babies, 16 had a simple noncritical CHD like VSD, ASD, and BAV, and 93 had a normal nonrevealing cardiac echo. CCHD was not observed in any of these babies.

Overall, the incidence of CHD among our term babies, who passed the POS within the first 24 hours of age, is estimated at 1.77% (16/900). This validates the limited ability of POS to detect simple noncritical CHDs that are not associated with oxygen desaturation. The fact that none of our cohort of term babies was subsequently diagnosed with CCHD supports as well POS use in screening for CCHD. Congruently, the efficacy of POS in screening for ductal-dependent critical cardiac lesions has been endorsed by a multitude of large multicenter cohort studies [16–19]. For CCHD, a POS sensitivity of 60 to 87.5% was proposed by these studies. Nonetheless, Ewer et al. suggested that the sensitivity of POS for all CHDs, including simple ones, is estimated at 49.1% [16]. Moreover, Koppel et al. argued that POS is appropriate for the detection of CCHD associated with significant hemodynamic instability and desaturation. However, a less significant CCHD, resistive to POS detection, may be missed within the first 24 hours of life [19].

In short, a negative POS cannot rule out simple CHD. As observed in our cohort, none of our term babies had a positive POS or was later diagnosed with a CCHD. However, 16 babies were found to have a simple CHD. Hence, we postulate that POS is not highly sensitive for simple CHD screening in term well-babies admitted to the regular nursery and that the combination of neonatal physical examination and echocardiography is sensitive for detecting these defects.

During this same year, 69 pregnant women presented to our tertiary care center for fetal echocardiography. 48 fetuses had a normal fetal cardiac echo, and 21 were found to have a congenital cardiac defect (Figure 2). Among these 21 fetuses, 5 had CCHD, and 16 had a simple noncritical CHD. These fetuses were transferred to the NICU immediately after delivery. Moreover, only 10 were delivered at our center and admitted to our NICU. 38 fetuses were admitted to regular nursery owing to the absence of CHD on fetal echo. This makes the incidence of CHD among our cohort of term babies and fetuses 3.97% (37/931) instead of 1.77%. Around 57% of these defects were detected on prenatal CHD screening and confirmed using fetal echocardiography. The majority (86.5%) of them were simple noncritical CHD, and 13.5%% were CCHD. All babies with CCHD were diagnosed prenatally using fetal echocardiography.

Given all the above, we attribute the absence of CCHD in our cohort of term well-babies to the existence of an effective fetal ultrasound and prenatal CHD screening unit in our center. Fetuses with CCHD are often suspected to have a hemodynamically significant cardiac defect on a routine detailed obstetric ultrasound showing the four chambers and the outflow tracts of the heart. These fetuses are then referred to our prenatal CHD screening unit for further assessment. We postulate also that POS could have detected the presence of CCHD among most of these fetuses upon delivery. However, a delayed diagnosis of these missed cases may have resulted in undesired outcomes if they were to be delivered in centers without immediate access to pediatric cardiologist and cardiac intervention. This stresses on the importance of having an adequate fetal cardiac center and justifies the absence of positive POS in our term well-babies. Finally, we argue that POS is probably adequate for detecting CCHD; however, it is less sensitive for detecting simple hemodynamically nonsignificant CHD that can be easily missed in term well-babies. We and others (Riede et al. and Narvey et al. [20, 21]) suggest that POS sensitivity can be augmented by a well-performed thorough neonatal physical examination that involves proper cardiac auscultation and heart sound evaluation.

### 5.1. Strengths and Limitations

Our study is one of a few studies discussing the accuracy of POS in detecting CHD in term well-babies admitted to a regular nursery in a tertiary center with an active prenatal screening program. Around 1,000 babies and fetuses were included. Upon discharge from the hospital, babies are followed up either by pediatricians working within our center or within our referral base. However, our results might be affected by a potential loss of follow-up, or by moving away from the area. Subsequently, this may underestimate the computed incidence of CHD among our cohort of term babies and fetuses.

## 6. Conclusion

In brief, our hypothesis is congruent with what is already known about POS. This technique is deemed effective in screening for CCHD. However, its sensitivity in screening for simple noncritical CHD is limited; it can be enhanced by performing a proper neonatal cardiac examination. Furthermore, the implementation of a prenatal CHD screening unit in tertiary care centers remains the best method for detecting and managing CCHD in a timely manner. Our results show that the combination of a comprehensive prenatal screening, careful physical exam, and postnatal echocardiography, whenever indicated, improved the detection of CHD, particularly simple noncritical CHD. In the absence of such a comprehensive program, POS remains a cost-effective modality for detecting CCHD.

## Figures and Tables

**Table 1:** Distribution of patients as per pre-/post-ductal SpO_2_ at birth/24 hours of age.

	# of newborns (%)
*Pre-/post-ductal SpO_2_ at birth*	
100%	662 (73.55%)
99%	157 (17.44%)
98%	65 (7.22%)
95-97%	16 (1.77%)
*Pre-/post-ductal SpO_2_ at 24 hours of age*	
100%	684 (76%)
99%	146 (16.22%)
98%	56 (6.22%)
95-97%	14 (1.55%)

**Table 2:** Distribution of the babies who received a cardiac echo at birth or within the 1^st^ five years of life as per the reason.

	# of newborns (%)
*Reason for cardiac echo (before discharge)*	
Murmur	42 (75%)
Abnormal fetal ECG	1 (1.78%)
Single umbilical artery	1 (1.78%)
Single palmar crease	1 (1.78%)
Family history of CHD	3 (5.35%)
Echogenic focus noted on fetal ultrasound	3 (5.35%)
Other	5 (8.92%)
*Reason for cardiac echo (within the 1^st^ five years of life)*	
Murmur	33 (62.26%)
Fatigue	1 (1.88%)
Cyanosis	4 (7.54%)
Suspected Kawasaki	3 (5.66%)
Family history of CHD	1 (1.88%)
Vasovagal	1 (1.88%)
Suspected perimyocarditis/endocarditis	2 (3.77%)
Screening due to undocumented reason	2 (3.77%)
Other	6 (11.3%)

**Table 3:** The echocardiography findings observed in groups A, B, and C, respectively. Group A consists of newborns who received cardiac echo at birth before discharge from regular nursery. Group B represents those who received an echo within the 1^st^ five years of life. Group C represents the fetuses who received prenatal CHD screening.

	# of newborns
*Cardiac echo findings in group A*	
Atrial septal defect (ASD)	2
Bicuspid aortic valve (BAV)	1
Ventricular septal defect (VSD)	7
Normal	46
*Cardiac echo findings in group B*	
ASD	6
Normal	47
*Cardiac echo findings in group C*	
Mitral atresia, single ventricle, and hypoplastic aorta	1
Apical displacement of tricuspid valve	1
ASD, coarctation of aorta, and VSD	1
Atrioventricular canal defect	3
BAV	1
D-transposition of the great arteries (D-TGA)	1
Ebstein anomaly	3
Hypoplastic left heart syndrome (HLHS)	1
Severe mitral regurgitation and total anomalous pulmonary venous return	1
Tetralogy of Fallot	3
Total anomalous pulmonary venous return (TAPVR)	1
Tricuspid atresia	1
Single VSD and malposed great arteries	1
VSD	2

## Data Availability

The data used to support the findings of this study are available from the corresponding author upon request.
